# Computational Evaluation of the Strict Master and Random Template Models of Endogenous Retrovirus Evolution

**DOI:** 10.1371/journal.pone.0162454

**Published:** 2016-09-20

**Authors:** Fabrícia F. Nascimento, Allen G. Rodrigo

**Affiliations:** National Evolutionary Synthesis Center, Durham, NC, United States of America; "INSERM", FRANCE

## Abstract

Transposable elements (TEs) are DNA sequences that are able to replicate and move within and between host genomes. Their mechanism of replication is also shared with endogenous retroviruses (ERVs), which are also a type of TE that represent an ancient retroviral infection within animal genomes. Two models have been proposed to explain TE proliferation in host genomes: the strict master model (SMM), and the random template (or transposon) model (TM). In SMM only a single copy of a given TE lineage is able to replicate, and all other genomic copies of TEs are derived from that master copy. In TM, any element of a given family is able to replicate in the host genome. In this paper, we simulated ERV phylogenetic trees under variations of SMM and TM. To test whether current phylogenetic programs can recover the simulated ERV phylogenies, DNA sequence alignments were simulated and maximum likelihood trees were reconstructed and compared to the simulated phylogenies. Results indicate that visual inspection of phylogenetic trees alone can be misleading. However, if a set of statistical summaries is calculated, we are able to distinguish between models with high accuracy by using a data mining algorithm that we introduce here. We also demonstrate the use of our data mining algorithm with empirical data for the porcine endogenous retrovirus (PERV), an ERV that is able to replicate in human and pig cells *in vitro*.

## Introduction

Transposable elements (TEs) are DNA sequences able to move and replicate within, and occasionally between, host genomes [1]. These elements are present in almost all species including prokaryotes, and they are believed to constitute more than half of the human genome [1–5]. TEs are classified in two main groups. Class I elements, or retrotransposons, replicate through an RNA intermediate and insert a DNA copy into a new locus in the host genome, moving by a “copy and paste” mechanism [6]. In contrast, class II elements or DNA transposons replicate mainly by a “cut and paste” mechanism, excising themselves from one locus and reinserting in a different location in the host genome [6]. Given the very different mechanism of replication between the two TE classes, this study focuses only on the class I elements or retrotransposons.

**Fig 1:**
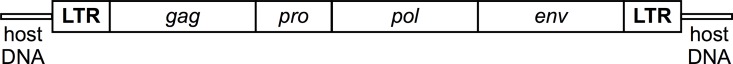
A schematic illustration of a provirus genome. The four main genes are depicted: *gag*, *pro*, *pol* and *env* genes. Proviruses are flaked by long terminal repeats (LTRs).

The retrotransposon mechanism of replication is also shared with retroviruses [6]. Retroviruses have a dimer positive sense and single stranded RNA genome [7, 8], which is organized in four main coding domains: *gag* (encoding capsid, matrix and nucleocapsid proteins), *pro* (protease), *pol* (reverse transcriptase and integrase enzymes), and *env* (surface and transmembrane glycoproteins of the virus envelope) genes [8, 9] (Fig 1). While simple retroviruses are composed of these main genes, complex retroviruses have additional accessory genes [9]. Once a retrovirus integrates in the host genome it is referred to as a provirus. Proviruses have two long terminal repeat (LTR) sequences flanking their genome and containing regulatory elements [9, 10] (Fig 1). These LTR sequences are identical at the moment of integration, and often these two sequences recombine forming a solo LTR [7, 11].

Although retroviruses usually infect somatic cells, they can also infect and colonize germ cells [9]. Proviruses in germ cells are known as endogenous retroviruses (ERVs) [12–14]. ERVs are viewed as an ancient retroviral infection in animal genomes and are commonly referred to as viral “fossils” [8, 15, 16]. They are present in multiple copies; are passed to the offspring; and account for approximately 8% of the human genome [9, 17, 18]. Although the majority of ERVs observed in host genomes are classified as simple retroviruses, there are reports showing the integration of complex retroviruses in germ line cells [19, 20]. Because proviruses have the potential to disrupt host gene expression, they are negatively selected and typically lose their viral function and ability to reinfect [9]. However, some ERVs are still able to reinfect because they have intact viral genes; examples of ERVs that can reinfect are the koala retrovirus (KoRV) [21], the porcine endogenous retrovirus (PERV) [22], and the cervid endogenous retrovirus (CrERV) [23]. Even though ERVs are usually negatively selected, it is clear that ERV and other TEs play a role in shaping host genomes [14, 24, 25]. The most striking example is the role of an ERV gene in the formation of the placenta in mammals [26, 27]. Recently, Chuong *et al*. [28] have also shown the importance of human ERV (HERV) sequences for the innate immune response.

LTR sequences contain binding sites for cellular transcription factors that aim to promote the transcription of the provirus [7, 29]. An ERV is initially transcribed by the host polymerase, but it will increase in copy number following either reinfection or retrotransposition [29–31]. Reinfection involves the release of a virus that will reinfect another cell, a process that requires intact copies of all viral genes [30, 32]. Retrotransposition is the proliferation of a virus without the requirement of reinfecting another cell, and can occur either in *cis* or as complementation in *trans* [30]. Retrotransposition in *cis* requires functional *gag* and *pol* genes, while for complementation in *trans* no functional genes are required [31, 32]. In the latter case, the ERV needs to have an intact LTR for initial viral transcription to occur, with the other proteins necessary for viral replication provided by other viruses or TEs [30, 31]. In this case, a genome of a defective ERV will be integrated to the host genome if it successfully packages a reverse transcriptase and integrase enzymes [30]. An example of such process is the HERV-W that has used proteins from long interspaced elements (LINEs) to retrotranspose [33].

Two models of class I retrotransposition have been described: the strict master model (SMM) and the random template (or transposon) model (TM). In SMM, it is assumed that only one element of a given lineage in the genome–the “master”–is capable of producing a new copy, while in TM, it is assumed that all elements of a given lineage in the genome are equally able to produce new copies [34, 35]. Clough *et al*. [35] described the expected phylogenetic tree topology of retrotransposons under these two models, but did not investigate whether current phylogenetic methods would recover the expected tree topologies.

Because of differences in genetic diversity, size, internal structure, and impact in host disease, it is important to understand the evolutionary dynamics of retrotransposons. Finding *in silico* models and evolutionary analyses that best explain their dynamics will advance our understanding of retrotransposons and their ability to retrotranspose in host genomes. In this paper, we focused on exploring these aspects by simulating SMMs and TMs on a type of class I elements or retrotransposons, the ERV. Our work is an extension of the work proposed by Clough *at al*. [35], but we include in our models ERV inactivation and ongoing activity related to their ability to retrotranspose or reinfect host cell genomes. Based on this information of ERV inactivation and ongoing activity, variations of SMM and TM were accessed in this study and named “SMM Mortal (SMM-m)”, “TM Mortal (TM-m)”, “SMM Immortal (SMM-i)” and “TM Immortal (TM-i)”. These four extreme models were chosen to investigate whether a maximum likelihood approach would recover the expected tree topologies under the SMM and TM as described by Clough *et al*. [35].

Our results show that one is more likely to recover trees similar to the expected phylogenetic trees when phylogenies were reconstructed using alignments of 10,000 base pairs (bp) rather than 1,000 bp. In general, it was also more likely to recover the expected topologies when the rate of ERV replication per host generation was low. By increasing the rate of ERV replication per host generation, it also became more difficult to distinguish tree topologies under SMM and TM. Nonetheless, when appropriate statistics were calculated for phylogenetic trees, we were able to correctly identify 84% and 93% of the different models when trees were reconstructed with alignments of 1,000 bp and 10,000 bp, respectively. Our statistical approach was also able to recover the expected replication patterns for porcine endogenous retroviruses (PERVs).

Our study showed the importance of thoroughly analyzing extreme models of ERV dynamics and evolution before more complex models could be proposed. For example, a more complex model could involve only a proportion of elements able to replicate in a host genome. If we were unable to correctly identify the models herein proposed, it would be unlikely to do so by using more complex models of ERV dynamics.

## Materials and Methods

### The models

We assume that a single exogenous retrovirus colonizes a host germ cell genome and simulations start from this single copy. This was considered the initial time in all simulations, and it was also the time this retrovirus is endogenized. One simulation run represented the evolution of a single ERV lineage.

We have applied to ERVs both SMMs and TMs [34, 35] generally described for TE replication in host genomes. SMMs assumes that only one element of a given lineage–the “master”–is able of producing a new copy, while TMs assumes that all elements of a given lineage are equally able to produce new copies in the host genome. Based on SMMs and TMs four models were assessed in this study and named “SMM Mortal (SMM-m)”, “TM Mortal (TM-m)”, “SMM Immortal (SMM-i)” and “TM Immortal (TM-i)”.

For the Mortal models, it was assumed that replication of an ERV lineage stopped after a fixed number of copies in the genome were attained. This follows the biological assumption that full-length ERVs are unable to reinfect when, for example, mutations cause gene inactivation of all ERV genes in all copies [9, 11]. A fixed number of elements was used based on information of copy number of ERV lineages in different host genomes [36]. In contrast, for the Immortal models, it was assumed that (*i*) an ERV lineage was able to replicate indefinitely and, (*ii*) the newly generated copy could occupy the locus of a previous copy by replacement. This follows the biological observation of full-length ERVs, such as the porcine endogenous retrovirus (PERV) [22] and the koala retrovirus (KoRV) [21], which still have the ability to reinfect or retrotranspose.

In our models, we do not distinguish between retrotransposition and reinfection. We assume that a newly generated ERV will be successfully reinserted in the host genome and become fixed, unless it is replaced as for the Immortal models.

### Computer Simulations

#### 1. Simulation of true phylogenetic trees

Computer simulations were carried out using two variables. The first variable was the ERV mutation rate (*μ*_*erv*_) set to 1.0×10^−4^, 1.0×10^−5^, 3.0×10^−5^, 1.0×10^−6^, 1.0×10^−7^, and 1.2×10^−8^ substitutions per nucleotide per infection (*s/n/i*). A mutation rate of 3.0×10^−5^ *s/n/i* has been estimated for Murine Leukemia Virus (MLV) [37], a virus that can be found in both exogenous and endogenous form [38]. We used other four ERV mutation rates to test the influence of this parameter on phylogenetic tree branch lengths. The second variable was the rate of ERV retrotransposition or reinfection per host generation that in this paper will be solely referred to as ERV replication (λ). No accurate information is available for ERV replication in host genomes. For this reason, arbitrary values of ERV replication were used in our simulations and were set to 1.0×10^−4^, 2.0×10^−4^, 3.0×10^−4^, 4.0×10^−4^, 5.0×10^−4^, 6.0×10^−4^, 7.0×10^−4^, 8.0×10^−4^, and 9.0×10^−4^ retrotranspositions or reinfections per host generation (*r/g*). Host substitution rate (*μ*_*h*_) was fixed at 1.2×10^−8^ substitutions per nucleotide per host generation (*s/n/g*), which is the described substitution rate for humans [39].

Because two mutation rates were used to simulate phylogenetic trees, branch lengths in substitutions per site represented a composite rate between host and ERV mutation rates. At least two different mutation rates are associated with the evolution of an ERV lineage [15, 40]. First, a new ERV copy will be the consequence of retrotransposition or reinfection because both mechanisms involve the reverse transcription of a viral RNA intermediate by the ERV encoded reverse transcriptase enzyme. Second, an ERV lineage can replicate because of host DNA replications by its DNA polymerase. Retroviral reverse transcriptase has a higher substitution rate than the host DNA polymerase [37, 40, 41].

The waiting time for an ERV to release a new copy in the host genome was simulated as an exponential random variable, with rate λ for SMMs and Nλ for TMs, where λ is the rate of ERV replication per host generation; and *N* is the number of ERV copies already generated. The ERV chosen to release a new copy in the host genome will release this new copy under the viral mutation rate (*μ*_*erv*_), while all other copies that remained in the genome will accumulate mutations according to the host substitution rate (*μ*_*h*_).

Different ERV lineages show different copy number, e.g. [36]. Because we are simulating simpler models to understand the dynamics of ERVs, the maximum number of ERV copies in the phylogenetic tree (*n*_*max*_) was set to 50. This number was chosen based on the copy number described for porcine endogenous retroviruses (PERVs) [42] and some human endogenous retroviruses (HERVs) [36]. Finally, 100 simulations were carried out for each combination of ERV mutation rate and ERV replication for a total of 1,000,000 host generations. We refer to simulated phylogenetic trees as true trees.

#### 2. Algorithm to simulate true phylogenetic trees for Mortal models

(1) A waiting time (*t*) is generated, which represents the time in host generations before an ERV releases a new copy in the host genome.

$$t=\frac{-ln(U)}{N\lambda }$$

Where *U* is uniform random number, *N* is the number of elements that is able to generate a new copy in the host genome (for SMM, *N* is always 1), and λ is the ERV replication rate per host generation.

(2) If the sum of waiting times is less than the maximum number of host generations (1,000,000), a new element will be added to the phylogenetic tree.

(3) If the total number of elements is less than the maximum number of elements (*n*_max_), branch lengths (*l*) will be calculated as follows:

$$l=\mu_{h}\times t$$

$$l_{nc}=(\mu_{h}\times t)+\mu_{erv}$$

where *l* and *l*_*nc*_ are the branch lengths of genomic ERVs and the new ERV, respectively.

(4) If the total number of elements is equal to the maximum number of elements, all copies will accumulate mutations according to the host mutation rate, and the final branch length (*l*_*f*_) will be calculated as follow:

$$T_{f}=T-\sum t$$

$$l_{f}=\mu_{h}\times T_{f}$$

where *T* is the maximum number of host generations, and *T*_*f*_ is the time at which the phylogenetic tree was composed by 50 elements (the maximum number of elements).

(5) If number of elements in the tree is less than the maximum number of elements, and the sum of *t* is less than the maximum number of host generations, then return to Step 1, otherwise STOP.

#### 3. Algorithm to simulate true phylogenetic trees for Immortal models

Steps (1), (2), and (3) are the same as described for the Mortal models.

(4) For Immortal models, replacement of elements is allowed. Replacement represents a homologous recombination between two proviruses [11]. The probability a replacement (*R*) will occur was calculated as:

$$P(R)=\frac{n-1}{n_{max}-1}$$

Where *n* represents the current number of elements (or number of tips) in the phylogenetic tree. The randomly chosen ERV that will give birth to a new element was not allowed to be replaced. For this reason, we subtract 1 element from the equation above. This probability was chosen following the biological assumption that as the number of elements in the tree increases, the probability of replacement also increases.

(5) Repeat Steps 3 and 4 until the sum of *t* reaches the maximum number of host generations.

#### 4. Simulation of DNA sequence alignments and phylogenetic reconstructions

Seq-Gen 1.3.3 [43] was used to simulate DNA sequence alignments of 1,000, 10,000, and 100,000 bp under the Jukes and Cantor (JC) substitution model [44] for each true tree generated under the four ERV models. The approximate size of an ERV genome is 10,000 bp [9]. However, it is common to reconstruct ERV phylogenies using partial genes of approximately 1,000 bp [45–48]. Simulations using 100,000 bp alignments were carried out to understand the effect of sampling errors in reconstructing ERV phylogenies.

Finally, using the simulated DNA sequence alignments, phylogenetic trees were reconstructed by maximum-likelihood (ML) with RAxML 8.0.19 [49] and setting the nucleotide substitution model to general time reversible (GTR) [50–52] (estimated values). Trees were rooted with a midpoint root using a script in R 3.0.3 [53] and the package *phangorn* [54]. Reconstructed phylogenetic trees will be referred to as ML trees.

### Statistical analysis

To compare true with ML trees reconstructed with alignments of 1,000, 10,000 and 100,000 bp, we used the Robinson-Foulds (RF) metric [55, 56] in the R package *phangorn*. Because RF metric is a partition metric, its range is 0 for identical trees with a maximum value of 2*n* – 6, where *n* is the total number of tips (or number of elements) in the tree [55]. The RF metric was calculated for rooted and unrooted trees to study the effect of midpoint rooting in ML trees. Comparison using ML trees reconstructed with alignments of 100,000 bp were carried out to understand the effects of sampling error in reconstructing the evolution of ERVs following the four proposed models.

Because the ERV genome size is approximately 10,000 bp, and because we would like to understand whether it is possible to distinguish phylogenies under different ERV models, tree statistics were calculated only for ML trees reconstructed using alignments of 1,000 and 10,000 bp. ML trees reconstructed using alignments of 100,000 bp were used solely to understand the effect of sampling error in reconstructing ERV phylogenetic trees.

The following 10 statistics were calculated as candidate variables for model classification, allowing us to test the best combination of statistics that is able to predict the correct ERV model proposed in this study:

(*i*) The tree shape statistic beta-splitting model (Beta) [57] was calculated using the R package *apTreeshape* [58]. Beta values equal to −2 represent completely unbalanced trees, which is expected for phylogenetic trees simulated under SMMs [35]. Increasing values of Beta correspond to greater tree balance [57], which is expected for phylogenetic trees simulated under TMs [35].

The other following seven tree shape statistics (*ii* to *viii*) were calculated using the R package *phyloTop* [59] following Colijn and Gardy [60], in which definitions are summarized below. For further information on statistics *ii* to *vii*, please see Colijn and Gardy [60].

(*ii*) Ladder length is defined by the maximum number of connected internal branches with a single terminal descendant branch (Max. ladder);

(*iii*) “IL” branches are defined as the portion of internal branches with a single terminal branch as descendant (“IL” portion);

(*iv*) Maximum depth and (*v*) maximum width: The depth of a branch is defined as the number of branches between that branch and the tree’s root, while the tree width at depth *d* is defined as the number of branches with depth *d;*

(*vi*) Maximum width over maximum depth: The ratio between maximum width and maximum depth;

(*vii*) Maximum difference in widths is defined as the maximum absolute difference in widths from one depth to the next, over all depths in the tree;

(*viii*) Number of cherries was also calculated; a cherry is defined as a pair of terminal branches that are adjacent to a common ancestor node [61].

To account for tree size, values obtained for summaries *ii*, *iii*, *iv*, *v*, *vii* and *viii* were divided by the number of terminal branches (or number of elements) in the tree.

In addition to tree shape statistics, (*ix*) the proportion of terminal branch lengths that contributed to the total tree branch length (“prop”) was calculated using an R script and the *ape* package [62]. A higher proportion is expected for the Mortal models. Finally, (*x*) nucleotide diversity for simulated DNA sequences was calculated for alignments of 1,000 bp and 10,000 bp also using an R script and the *pegas* package [63].

### Comparison of true and ML trees and classification of ERV models

To compare the distribution of each of the 10 statistics calculated in the previous section for SMM and TM, we used the Jensen-Shannon divergence (JSD). The JSD is a symmetric divergence statistic that can be used to measure similarities between two distributions [64]. Comparisons were performed in pairs for SMM-m and TM-m as well as SMM-i and TM-i: JSD was calculated for true trees as well as for ML trees for each statistics described in the previous section. If two distributions are identical JSD = 0, and larger values of JSD represents dissimilar distributions. In the context of this study, if JSD = 0 there is no difference between trees under SMM and TM.

Because of the different tree topologies expected for trees generated under the SMM and TM [35], we would expect larger values for JSD calculated for the true trees under SMM and TMs for each of the tree shape statistics. With finite sequence data, we expect that errors in phylogenetic reconstruction will introduce variation in the differences of JSD statistics.

Because we would like to distinguish between trees reconstructed under the SMM and TM, we calculated JSD for each statistics for all combination of models in pairs (for example, TM-m *vs* TM-i, SMM-m *vs* TM-i, *etc*) using ML trees. We chose the statistics in which the JSD was larger and different from zero. This was used as a pre-screening of which variables should be included in a *k*-nearest neighbor (*k*NN) classifier. We also trained a *k*NN classifier using only the Beta statistics, which is a metric to detect phylogenetic tree imbalance.

A *k*NN was trained using R and the function IBk of package RWeka [65]. We let the function automatically find the best number of nearest neighbor value *k* between 1 and 30. We also tested *k* varying between 1 and 100. This training was performed on values of tree statistics and nucleotide diversity calculated for 21,600 ML trees / DNA sequence alignments of 1,000 and 21,600 ML trees / DNA sequence alignments of 10,000 bp. Results are reported using a 10-fold cross-validation and the *k*NN classifier trained with alignments of 1,000 bp were cross-validated using only 1,000 bp alignments. Similarly, a *k*NN classifier trained with alignments of 10,000 bp was cross-validated using only 10,000 bp alignments.

There is a lack of information regarding ERV model of replication (SMM or TM), ERV replication rate per host generation and ERV mutation rate. For this reason, and because few ERVs are know to be able to replicate in host genomes, we decided to train the *k*NN to classify between the four proposed models rather than trying to improve performance by classifying between SMM-m/TM-m and between SMM-i/TM-i, for example.

### Empirical data

We used datasets of PERV DNA sequences from two different lineages of PERVs, the gamma1 and gamma2 PERVs. Because of polymorphism in their *env* gene, gamma 1 PERVs is further divided into A, B and C classes [22, 66, 67], while gamma2 PERVs comprises only PERV class E [68, 69]. While gamma1 has the ability to replicate [22, 70], this does not seem to be the case for gamma2 PERVs [47, 68, 69]. To demonstrate how the framework developed in this paper could be used for empirical data, we analyzed 46 sequences comprising genomic data for *Sus scrofa* gamma1 PERVs (classes A, B and C), with an alignment of 9,017 bp (including gaps). Because some alignment differences were observed between PERV-A, -B and -C, we also analyzed a subset of 24 sequences comprising only PERV class B. PERV-B alignment comprised 8,762 bp (including gaps). From those 46 genomic data, we also analyzed 1,000 bp of the *pol* gene. Finally, we analyzed 999 bp of 50 sequences of *env* type E for gamma 2 PERVs in *Sus* species.

Sequences were obtained from GenBank (for accession numbers see alignments at GitHub. Information is available in Code and Data availability section), and to increase sample size, PERV genomic sequences were also mined from the *Sus scrofa* genome (version Sscrofa 10.2) using blastn. All sequences were aligned using Muscle [71] with default options implemented in the program seaview [72]. Alignments were manually curated according to Yang [73].

Phylogenetic trees were reconstructed using the same methodology as described for simulated DNA sequences and using the GTR + Γ [50–52] as the DNA substitution model. The same statistics for tree shape, nucleotide diversity and proportion of terminal branch lengths that contributed to the total tree branch length were calculated using the same approach described for simulated data. We used the *k*NN algorithm trained with ML trees reconstructed with 1,000 bp and 10,000 bp to make predictions using partial gene and genomic sequence data, respectively.

### Code and Data availability

Algorithms to simulate the four ERV models described in this paper were written in Python and used the Python package ETE2 [74] to simulate rooted phylogenetic trees with branch lengths. This Python code is available at https://github.com/thednainus/ERV_Simulations

A pipeline in R to calculate the same statistics for empirical phylogenetic trees is available at https://github.com/thednainus/R_Pipeline. The *k*NN classifiers trained with reconstructed phylogenetic trees and DNA sequences alignments of 1,000 bp and 10,000 bp can also be downloaded for future predictions of the proposed models described in this paper.

Sequence alignments for porcine endogenous retrovirus used in this study can be downloaded at https://github.com/thednainus/R_Pipeline/tree/master/alignments. Information on GenBank accession numbers can also be found in these alignments.

## Results

A total of 21,600 trees were simulated following the different ERV models proposed in this study (see Materials and Methods). The maximum number of elements or tips (*n*_*max*_ = 50) per phylogenetic tree was achieved in all simulations with the exception of simulations following the SMM-i. In this case, *n*_*max*_ = 50 was achieved when ERV replication was set to 6.0×10^−4^, 7.0×10^−4^, 8.0×10^−4^, and 9.0×10^−4^ retrotranspositions or reinfections per host generation (*r/g*). For other ERV replication variables, the total number of elements or tips (*n*) in the phylogenetic tree ranged from 36 to 50 elements, with the majority of observations between 48 to 50 elements (Table 1).

**Table 1:** Frequency table showing the number of elements or tips (*n*) observed for each phylogenetic tree following the SMM-i when ERV replication was set to 1.0×10^−4^ to 5.0×10^−4^.

	*n*														
ERV replication	36	37	38	39	40	41	42	43	44	45	46	47	48	49	50
1.0×10^−4^	1	3	10	13	39	53	78	89	100	95	58	40	16	5	0
2.0×10^−4^	0	0	0	0	0	0	0	0	0	2	3	23	97	214	261
3.0×10^−4^	0	0	0	0	0	0	0	0	0	0	0	0	5	60	535
4.0×10^−4^	0	0	0	0	0	0	0	0	0	0	0	0	0	5	595
5.0×10^−4^	0	0	0	0	0	0	0	0	0	0	0	0	0	3	597

Unsurprisingly, the agreement between true and estimated phylogenies improved as sequence length increased: Maximum likelihood (ML) trees reconstructed using longer alignments of 100,000 bp had the lowest Robinson-Foulds (RF) distance [55, 56] to the true trees, followed by ML trees reconstructed using alignments of 10,000 bp and lastly, by ML trees from 1,000 bp alignments (S1–S3 Figs). In general, using the midpoint root on reconstructed trees was sufficiently robust; no strong difference was observed between true and ML trees when different ERV mutation rates were considered (S1–S3 Figs). However, as the rate of ERV replication increased, it became more difficult to reconstruct trees similar to the true trees (S1–S3 Figs).

**Fig 2:**
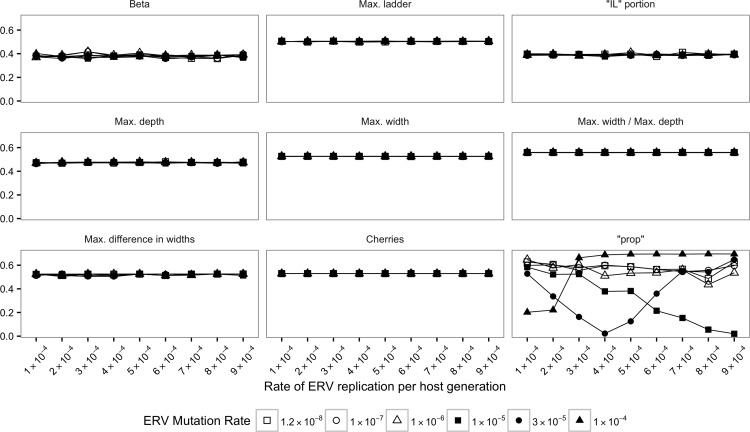
Jensen-Shannon divergences (JSDs) between SMM-m and TM-m for true trees. Each plot and its y-axis represent a statistic summary. ERV replication rate per host generation is depicted in the x-axis.

**Fig 3:**
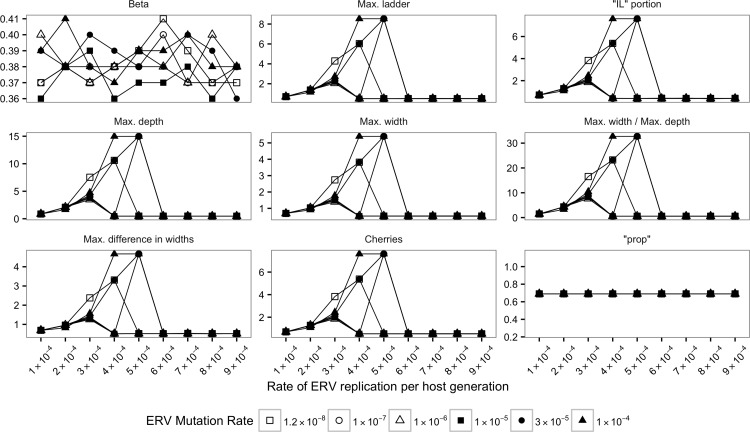
Jensen-Shannon divergences (JSDs) between SMM-i and TM-i for true trees. Each plot and its y-axis represent a statistic summary. ERV replication rate per host generation is depicted in the x-axis.

**Fig 4:**
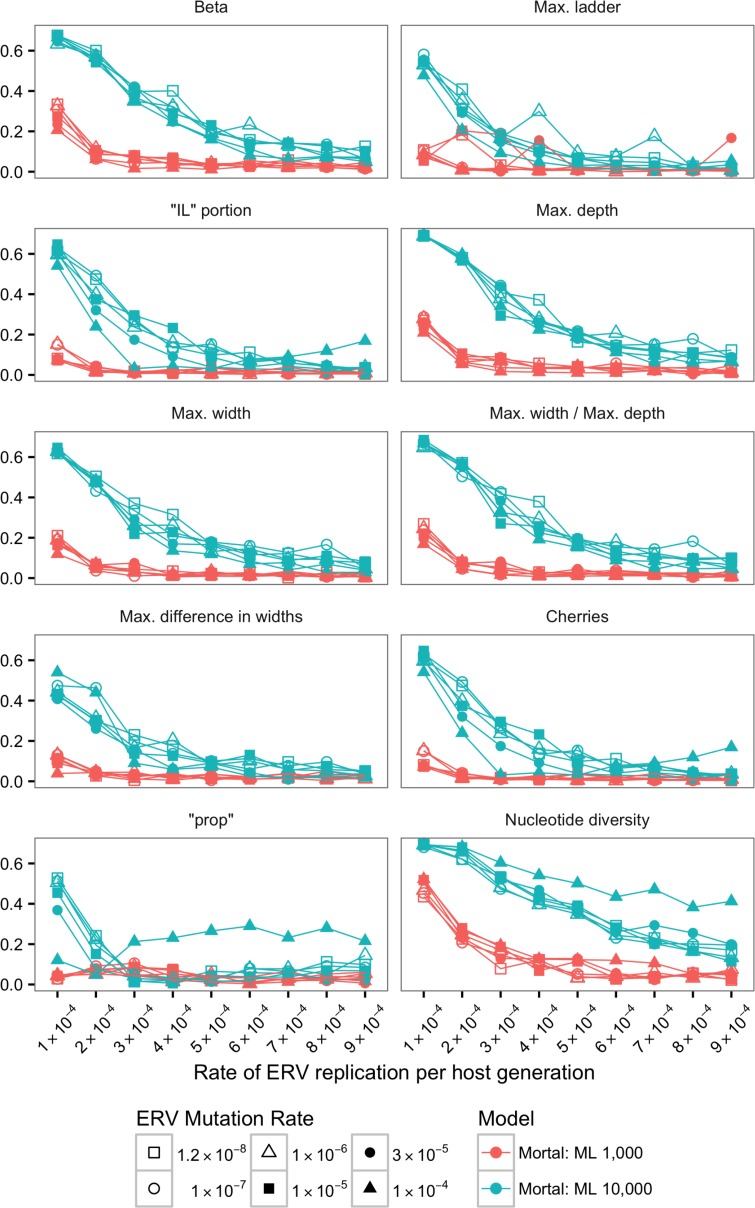
Jensen-Shannon divergences (JSDs) between SMM-m and TM-m for ML trees using alignments of 1,000 bp and 10,000 bp. Each plot and its y-axis represent a statistic summary. For all 10 plots, ERV replication rate per host generation is depicted in the x-axis.

**Fig 5:**
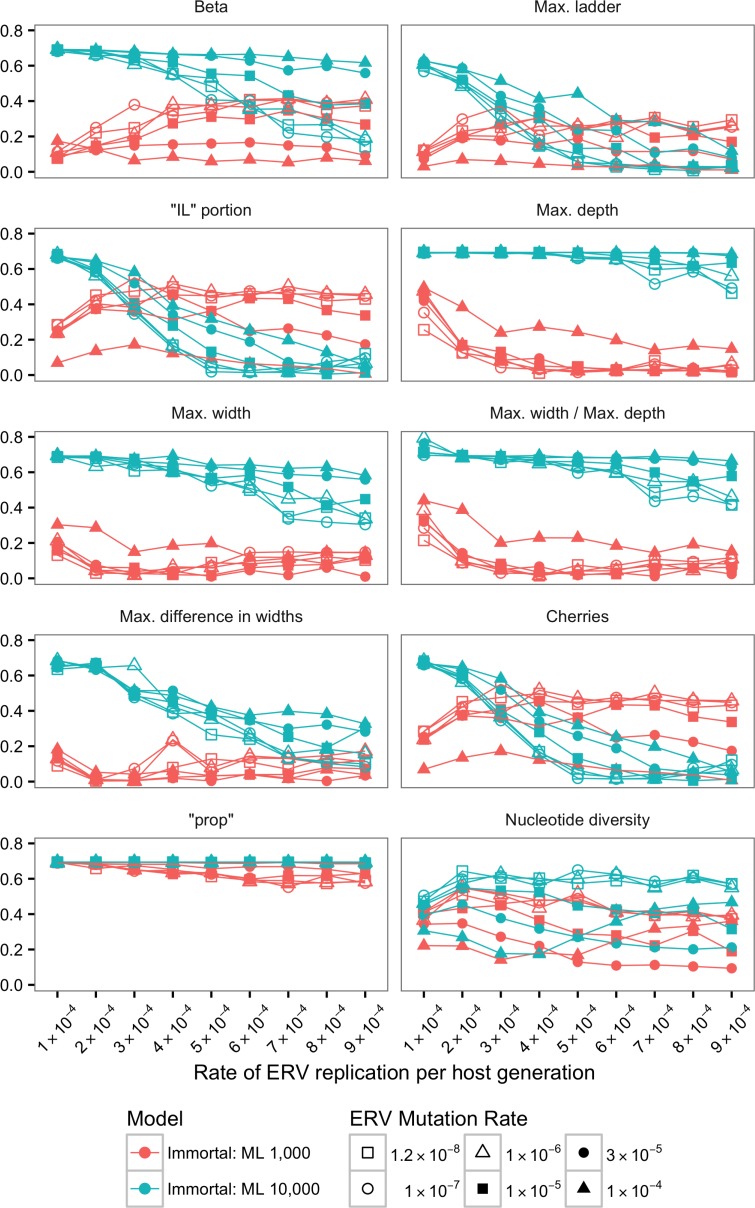
Jensen-Shannon divergences (JSDs) between SMM-i and TM-i for ML trees using alignments of 1,000 bp and 10,000 bp. Each plot and its y-axis represent a statistic summary. For all 10 plots, ERV replication rate per host generation is depicted in the x-axis.

The Jensen-Shannon divergence (JSD) was calculated for the distribution of each statistics (see Material and Methods) between SMM-m and TM-m as well as SMM-i and TM-i (Figs 2–5).

For almost all true trees, the values of JSD were higher than zero (Figs 2 and 3) suggesting very different distributions for SMM-m and TM-m as well as SMM-i and TM-i. Visual inspection of boxplots (data not shown) for the distribution of each statistics confirmed that it is possible to distinguish between SMM-m and TM-m and between SMM-i and TM-i.

An exception was JSD calculated between SMM-m and TM-m for the proportion of terminal branch lengths that contributed to the total tree branch length (“prop”) (Fig 2). In that case, when ERV mutation rate (*μ*_*erv*_) was 1.0×10^−5^ *s/n/i*, JSD tended to zero as the rate of ERV replication per host generation (λ) increased. When *μ*_*erv*_ = 3.0×10^−5^ *s/n/i*, JSD tended to zero until λ = 4.0×10^−4^ *r/g*. After this point JSD increased (Fig 2). Visual inspection of boxplots (data not shown) showed that for λ < 4.0×10^−4^ *r/g* the distribution of “prop” values obtained for TM-m are higher than those obtained for SMM-m. For λ > 4.0×10^−4^ *r/g*, the opposite was observed: the distribution of “prop” values obtained for SMM-m are higher than those obtained for TM-m. When λ = 4.0×10^−4^ *r/g*, the distribution of “prop” values obtained for TM-m and SMM-m were very similar. Finally, when *μ*_*erv*_ = 1.0×10^−4^ *s/n/i*, and λ ≤ 2.0×10^−4^ *r/g*, JSD was approximately 0.2. Visual inspection of boxplots (data not shown) showed some overlap of “prop” values. As ERV replication increased (λ > 2.0×10^−4^ *r/g*), JSD was higher than 0.6 showing that is possible to distinguish between SMM-m and TM-m (also confirmed by visual inspection of boxplots).

For ML trees, JSD calculated for the distribution of the 10 statistics (Figs 4 and 5) indicated that with long enough sequences it is possible to use shape statistics, “prop” and nucleotide diversity from ML trees to identify the underlying model of ERV evolution.

However, JSD values for tree shape statistics for ML trees reconstructed with alignments of 1,000 bp showed that in most cases it was difficult to distinguish between SMM-m and TM-m models (Fig 4) and between SMM-i and TM-i models (Fig 5). JSD for the distribution of tree shape statistics using alignments of 1,000 bp showed, in general, similar distributions for SMM-m and TM-m, suggested by values of JSD closer to zero. Similar pattern was observed for JSD calculated for SMM-i and TM-i and alignment of 1,000 bp. However, JSD for “IL” portion and number of cherries for immortal models and alignments of 1,000 bp suggested that it is possible to distinguish between SMM-i and TM-i (Fig 5).

In contrast, JSD values calculated for the distribution of tree shape statistics for ML trees reconstructed with alignments of 10,000 bp showed that it was possible to distinguish between SMM-m and TM-m models and between SMM-i and TM-i models when the rate of ERV replication was low (Figs 4 and 5). However, as this rate increased, it became, in general, more difficult to distinguish between SMM-m and TM-m models (Fig 4), and between SMM-i and TM-i models (Fig 5) as suggested by values of JSD approaching zero. However, JSD for max. depth, max. width, and max. width / max. depth for immortal models and alignments of 10,000 bp suggested that it was possible to distinguish between SMM-i and TM-i even when the rate of ERV replication was high.

JSD calculated for SMM-m and TM-m for ML trees reconstructed with alignments of 1,000 bp for “prop” (Fig 4) was close to zero for all ERV mutation and ERV replication rates analyzed, suggesting that it was not possible to distinguish between SMM-m and TM-m when using only “prop”. In contrast, JSD calculated for SMM-m and TM-m for ML trees reconstructed with alignments of 10,000 bp (Fig 4) suggests that it was possible to distinguish between the two mortal models when ERV replication rate was the lowest.

JSD calculated for SMM-i and TM-i for ML trees reconstructed with alignments of 1,000 bp (Fig 5) for “prop” showed higher values than those obtained for the mortal models (Fig 4), suggesting that it was possible to distinguish between SMM-i and TM-i (Fig 5).

Our simulations show that it is also possible to distinguish between Mortal and Immortal models by calculating the proportion of terminal branch lengths to total tree branch length: True trees for Mortal models had longer terminal branches than true trees for Immortal models. This was true even when ML trees were reconstructed with alignments of 1,000 bp. In this case, only a small overlap of values calculated for SMM-m and SMM-i models were observed (data not shown). Nucleotide diversity was also a good measure to distinguish between Mortal and Immortal models as a higher diversity was always observed for Mortal models.

### Classification of ERV models

Although the four ERV models used for simulations are very different and indeed statistical analysis of true trees showed distinct values, it was still possible to distinguish between phylogenetic trees simulated under a Master or a Transposon model. The same was not observed when analyzing ML trees. For ML trees, JSD was closer to zero (indicating similar distributions) for several combinations of ERV mutation rate and replication, and the use of a classification method to distinguish between the different models, in this case, is very useful. In this context, we developed a *k*-nearest neighbor (*k*NN) classifier using ML trees reconstructed with alignments of 1,000 bp and 10,000 bp to identify models of ERV evolution. The *k*NN classifier was trained with ML trees and not true trees, because our results show that was not possible to fully recover true trees using alignments of 1,000 bp or 10,000 bp.

Pre-screening of JSD of the 10 statistics (data not shown) between all possible combinations of models (for example, TM-m *vs* TM-i, SMM-m *vs* TM-i, etc.) was used to detect the best combination of variables to be used in a *k*NN classifier. We chose the statistics with JSD values higher than 0.6 for the majority of observations. This suggested that statistics Beta, “IL” portion, number of cherries, “prop” and nucleotide diversity should be used to construct a classifier using ML trees reconstructed with alignments of 1,000 bp. Similarly, statistics Beta, max. depth, “prop” and nucleotide diversity should be used to construct a classifier using ML trees reconstructed with alignments of 10,000 bp.

To let the function automatically find the best number of nearest neighbor (see Materials and Methods), two sets of values of *k* were tested: *k* ranging from 1 to 30, and *k* ranging from 1 to 100. Very similar results were obtained. Below we report results obtained when *k* varied from 1 to 30.

With 1,000 bp alignments, 28 nearest neighbors were used in the *k*NN classifier. Using this classifier to predict the model underlining our own ML trees reconstructed with 1,000 bp alignments, we were able to correctly classify 84.37% of the four ERV models assessed in this study (Table 2). No misclassification was observed between Mortal and Immortal models, and all SMM-m was correctly classified. A higher misclassification rate was observed between SMM-m and TM-m models than between SMM-i and TM-i models.

**Table 2:** Table showing the results of a *k*-nearest neighbor (*k*NN) classifier using maximum likelihood (ML) trees reconstructed with alignments of 1,000 bp and 10,000 bp. Precision is the proportion of the examples which truly have class x among all those which were classified as class x [75].

	**ML 1,000 bp**			**ML 10,000 bp**		
Correctly Classified Instances	18,226 (84.37%)			20,117 (93.13%)		
Incorrectly Classified Instances	3,374 (15.63%)			1,483 (6.87%)		
**ERV Model**	**TP Rate**^1^	**FP Rate**^2^	**Precision**	**TP Rate**^1^	**FP Rate**^2^	**Precision**
Master Immortal (SMM-i)	0.999	0.001	0.996	1.000	0.000	1.000
Master Mortal (SMM-m)	0.623	0.081	0.719	0.839	0.038	0.881
Transposon Immortal (TM-i)	0.996	0.000	1.000	1.000	0.000	1.000
Transposon Mortal (TM-m)	0.756	0.126	0.667	0.887	0.054	0.846

^1^ True Positive Rate

^2^ False Positive Rate

With 10,000 bp alignments, 12 nearest neighbors were used in the *k*NN classifier. Using this classifier to predict the model underlining our own ML trees reconstructed with 10,000 bp alignments, we were able to correctly classify 93.13% of all models (Table 2). No misclassification was observed between Mortal and Immortal models, and all SMM-i and all TM-i were correctly classified.

Using both classifiers mentioned above, it was difficult to classify SMM-m and TM-m models, but a better classification was achieved when training a *k*NN using data calculated for ML trees reconstructed with alignments of 10,000 bp (Table 2).

### Empirical data

All data analyzed for PERV gamma1 was classified as TM-i with a very high probability (99.9%). The *env* E gene of the gamma2 PERV was classified as TM-m also with a very high probability (92.8%).

## Discussion

Transposable elements make up a part of the large fraction of what is considered non-coding DNA in eukaryotic genomes [76, 77], and recent studies are showing the significant role of TEs in shaping host genomes by restructuring genes and providing new regulatory sequences [2, 78, 79]. Although TEs are considered as non-coding DNA in their host genomes, TEs can encode their own proteins responsible for their replication. TE-encoded proteins can be co-opted into functional proteins within the host in an evolutionary process referred to as “molecular domestication” [79]. The most interesting example of co-option between ERV and hosts is the use of the *env* ERV gene in the formation of the placenta in mammals, including primates, rodents, lagomorphs and marsupials [26, 27, 80]. TE mobility can also negatively affect the host, as they are associated with disease by insertional mutagenesis and homologous recombination [81, 82].

Several studies suggest that retrotransposons, including ERVs, replicate following a SMM or TM [35, 83, 84]. In these cases, phylogenetic tree topologies were used as a reliable indication to determine whether an ERV lineage replicates following one of these models. ERV lineages following a SMM always generate completely unbalanced phylogenetic trees, while those following a TM tend to generate more balanced trees [35]. Our results from analyzing true trees also confirm this previous study [35]. Even though our study confirms the phylogenetic trees expected for SMM and TM as described in Clough *et al*. [35], we demonstrated that to accurately reconstruct these trees is not possible either using alignments of 1,000 bp or 10,000 bp. Unsurprisingly, it was more difficult to reconstruct ML trees that were similar to the true trees with shorter sequences. Our analyses, using alignments of 1,000, 10,000 and 100,000 bp, show that a likely reason for not recovering the true tree when ML trees are reconstructed using alignments of 1,000 and 10,000 bp is sampling error induced by limited numbers of sites.

Because an ERV genome size is approximately 10,000 bp and because ERV genomes can be mined from publicly available host genomes, we focused on understanding whether ML trees reconstructed with alignments of 1,000 or 10,000 bp would be consistent with a SMM or TM. Even though it is difficult to distinguish between different models by visual inspection of ML trees, we were still able to classify with high accuracy the four ERV models proposed in this study using the *k*NN classifier and the summary statistics for ML trees reconstructed with alignments of 10,000 bp. Interestingly, we were also able to correctly classify ML trees reconstructed with alignments of 1,000 bp, although the false positive rate for the Mortal models was higher than that obtained using 10,000 bp alignments.

There is a great interest in knowing whether an ERV lineage is still able to proliferate in a host genome or whether this ability has been lost. The ability to proliferate involves several mechanisms from reinfection to retrotransposition in *cis* and complementation in *trans*. Reinfection can lead to cross-species transmission of ERVs, which have been documented and occurs more than previously thought [85]. Even though our models do not distinguish between these mechanisms or whether horizontal transmissions have occurred, our models can detect whether a retrotransposon is still able to retrotranspose to different loci in a group of closely related ERVs. New ERV integrations may have several consequences for the host, from beneficial to detrimental and these new integrations may disrupt a host gene or cause diseases [7, 13, 86]. Our results indicated that although it was difficult to distinguish between SMM-m and TM-m models, a total separation was achieved between Mortal and Immortal models when using ML trees reconstructed with alignments of either 1,000 bp or 10,000 bp. These results suggest that it is possible to understand whether an ERV lineage is still able to show ongoing activity by retrotransposing or reinfecting host cells, or whether it lost this ability a very long time ago. This would be a cheaper alternative to pre-screening for ERVs that can or not retrotranspose or reinfect.

Other explanations have also been proposed to describe how retrotransposons replicate in host genomes [34]. For example, there are suggestions that more than one master template exists for an ERV lineage; or during the course of an ERV lineage evolution, a master template may become inactive with another ERV copy occupying its position [45, 87]. In this paper, we focused on understanding whether it was possible to distinguish evolutionary patterns with simpler models before simulating more complex models. According to our results, we would expect that in cases where more than one master templates are present in an ERV lineage, ML trees would resemble those simulated under the simpler SMM models proposed in this study.

Our results were consistent in showing that visual inspection of phylogenetic trees, e.g. [45, 83], is not the appropriate method to decide whether an ERV lineage is replicating following a SMM or TM model, and it is likely that this result can be extended to any TE lineage with limited genome size. In addition, using only one metric to check tree imbalance–in this paper, the Beta statistic–is also not sufficient in distinguishing between the models proposed here. This was evaluated by training a classifier using only the Beta statistics. In this case, only 63.24% and 58.98% of the models were correctly classified when a *k*NN was trained with ML trees reconstructed with alignments of 1,000 bp and 10,000 bp, respectively. We suggest that for a better classification of models, the Beta, “IL” portion, number of cherries, “prop” and nucleotide diversity should be used in a classifier when using alignments of approximately 1,000 bp. Similarly, the Beta, max. depth, “prop” and nucleotide diversity should be used in a classifier when using alignments of approximately 10,000 bp. When a classifier trained using these statistics was used, we were able to correctly classify ≈84% and ≈93% of all models when using summary statistics calculated for ML trees reconstructed with alignments of 1,000 bp and 10,000 bp, respectively.

Analysis of empirical data using our approach suggested that gamma1 PERVs are still able to replicate. In fact, the ability of gamma1 PERVs to replicate in host genomes is supported by *in vitro* studies [22, 70]. On the other hand, analysis of gamma2 PERVs using our approach suggested that these ERVs may have lost this ability; again, genetic studies showing that these ERVs have several stop codons and frame-shift mutations in all their genes [68] are consistent with this conclusion. Expression analysis of gamma2 PERVs also showed an inconsistent pattern when different samples were analyzed corroborating to the hypothesis that gamma2 may not be replicating [47, 69]. Our analysis also indicated that gamma1 PERVs in *Sus scrofa* is possibly replicating in accordance with a TM-i, while gamma2 PERVs in *Sus* species are replicating following a TM-m.

## Conclusion

We confirmed a previous study [35] that SMM and TM show very distinct phylogenetic tree shape. However, we demonstrated for the first time that it is not possible to accurately reconstruct these true trees using either alignments of 1,000 bp or 10,000 bp. A likely reason for this was sampling errors induced by limited number of sites, as reconstruction of true trees using alignments of 100,000 bp showed the lowest Robinson-Foulds distance (S1–S3 Figs). Given that the size of an ERV genome is limited and approximately 10,000 bp, and based on information obtained in this study we developed a *k*NN classifier to predict the likely model of TE replication and evolution in host genomes.

We suggest that instead of visual inspection of phylogenetic tree as used in some studies, e.g. [45, 83], one should calculate the statistics proposed in this study and use the respective classifier to gain a better understanding of the underlying model of TE replication and evolution. This developed classifier could also be used to predict whether a retrotransposon lineage is still able to proliferate or lost this ability a long time ago.

Although the proposed models described in this study represent simplistic models of ERV replication and evolution. This study represents an important step to understand whether it is possible to reconstruct trees similar to the expected trees under the SMM and TM. With the development of a *k*NN classifier we were able to distinguish between models with high accuracy. If we were unable to predict whether phylogenetic trees were from a SMM or TM, it would be unlikely to do so using more complex models. This is because more complex models of ERV evolution would involve variations of the simplistic models we are analyzing in this study. Our results are promising for the future development of more complex models of ERV replication and evolution in host genomes.

## Supporting Information

S1 Fig Robinson-Foulds (RF) metric for true and ML trees reconstructed with alignments of 1,000 bp. Plots for each ERV mutation rate showing RF metric (y-axis) for the Strict Master (SMM) and Transposon (TM) mortal and immortal models for ERV replication per host generation (x-axis). RF metrics were calculated for rooted and unrooted trees comparing true phylogenetic trees with ML trees reconstructed using alignments of 1,000 bp.
(PDF)

S2 Fig Robinson-Foulds (RF) metric for true and ML trees reconstructed with alignments of 10,000 bp. Plots for each ERV mutation rate showing RF metric (y-axis) for the Strict Master (SMM) and Transposon (TM) mortal and immortal models for ERV replication per host generation (x-axis). RF metrics were calculated for rooted and unrooted trees comparing true phylogenetic trees with ML trees reconstructed using alignments of 10,000 bp.
(PDF)

S3 Fig Robinson-Foulds (RF) metric for true and ML trees reconstructed with alignments of 100,000 bp. Plots for each ERV mutation rate showing RF metric (y-axis) for the Strict Master (SMM) and Transposon (TM) mortal and immortal models for ERV replication per host generation (x-axis). RF metrics were calculated for rooted and unrooted trees comparing true phylogenetic trees with ML trees reconstructed using alignments of 100,000 bp.
(PDF)
