# Supplementary figures and images for: Computational Evaluation of the Strict Master and Random Template Models of Endogenous Retrovirus Evolution
Source: PLoS One. 2016 Sep 20;11(9):e0162454. doi: 10.1371/journal.pone.0162454 (PMC5029938; doi:10.1371/journal.pone.0162454)

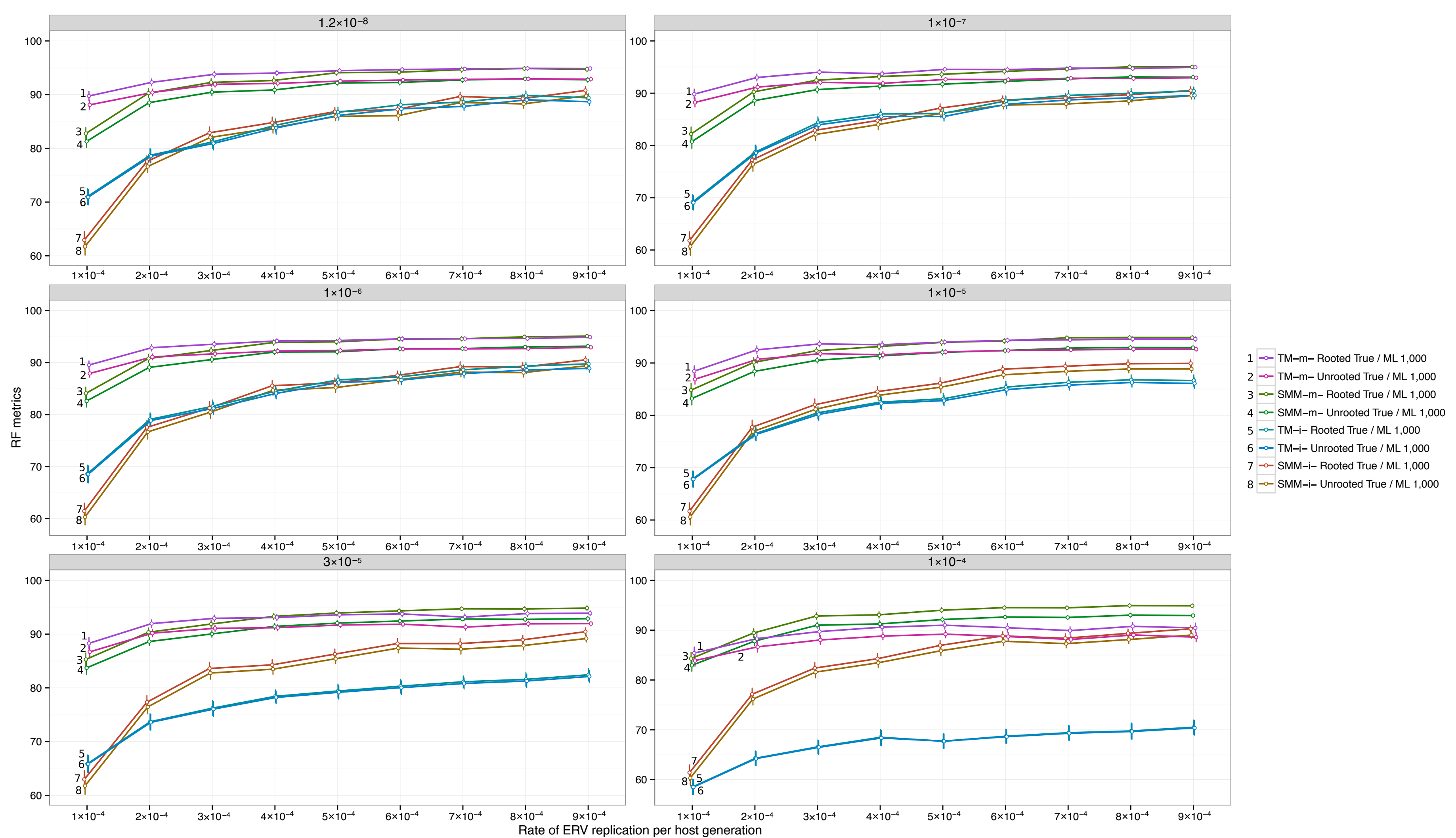

Supplement: S1 Fig — Plots for each ERV mutation rate showing RF metric (y-axis) for the Strict Master (SMM) and Transposon (TM) mortal and immortal models for ERV replication per host generation (x-axis). RF metrics were calculated for rooted and unrooted trees comparing true phylogenetic trees with ML trees reconstructed using alignments of 1,000 bp. (PDF) [file pone.0162454.s001.pdf]

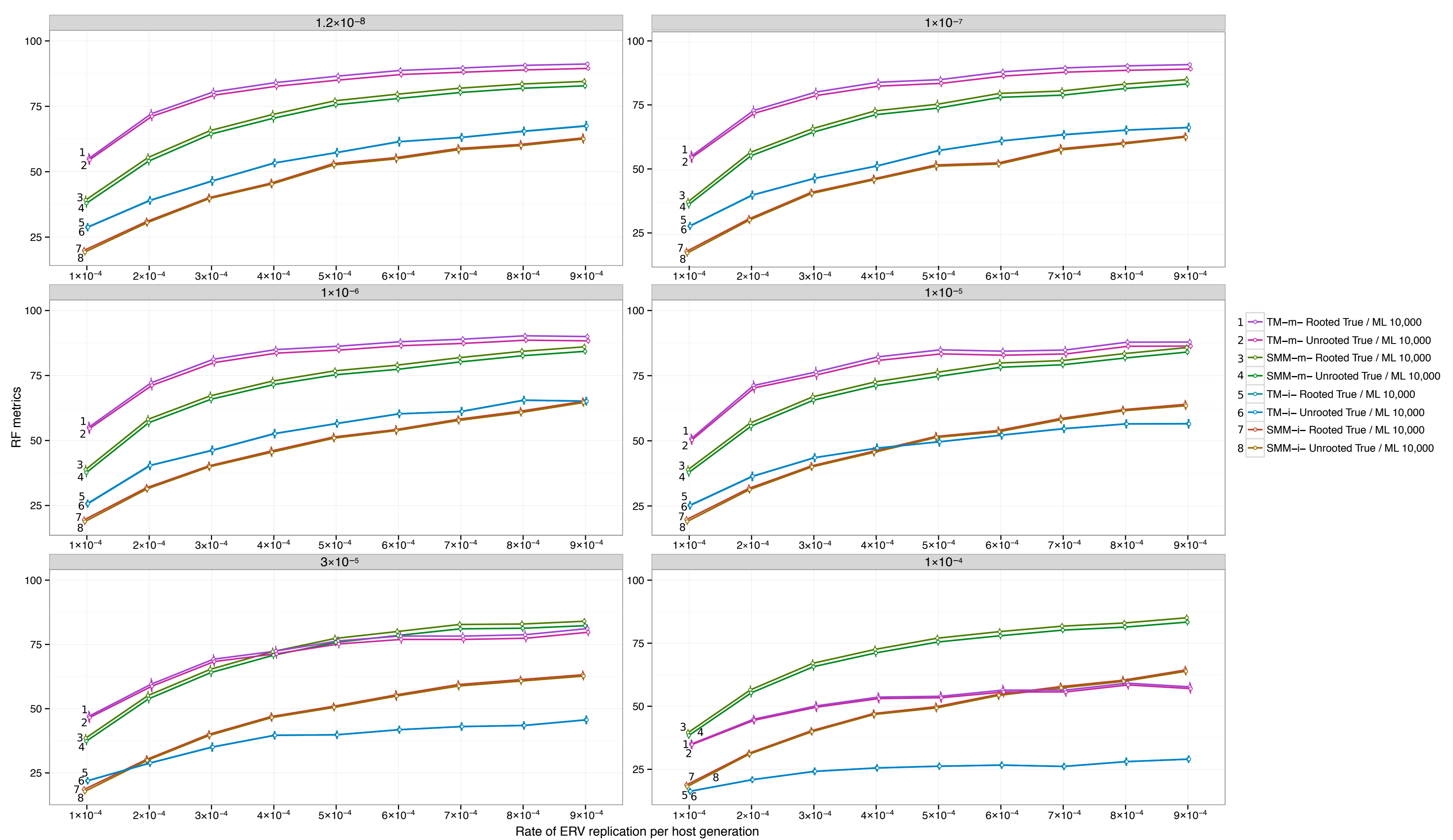

Supplement: S2 Fig — Plots for each ERV mutation rate showing RF metric (y-axis) for the Strict Master (SMM) and Transposon (TM) mortal and immortal models for ERV replication per host generation (x-axis). RF metrics were calculated for rooted and unrooted trees comparing true phylogenetic trees with ML trees reconstructed using alignments of 10,000 bp. (PDF) [file pone.0162454.s002.pdf]

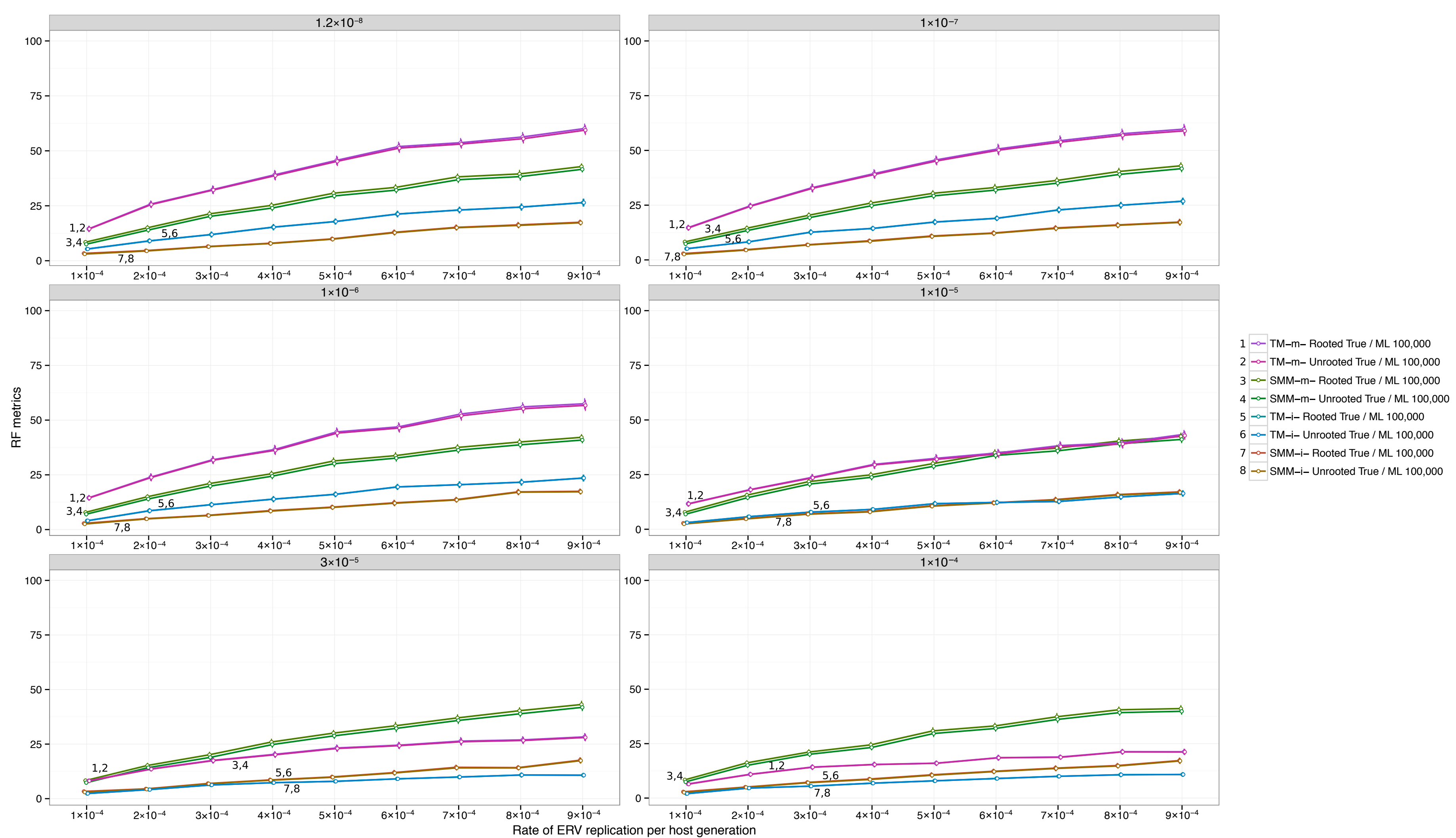

Supplement: S3 Fig — Plots for each ERV mutation rate showing RF metric (y-axis) for the Strict Master (SMM) and Transposon (TM) mortal and immortal models for ERV replication per host generation (x-axis). RF metrics were calculated for rooted and unrooted trees comparing true phylogenetic trees with ML trees reconstructed using alignments of 100,000 bp. (PDF) [file pone.0162454.s003.pdf]
